# Post-surgical Autopsy Findings: Insights Into Forensic Evaluation and Surgical Complications

**DOI:** 10.7759/cureus.95805

**Published:** 2025-10-31

**Authors:** G Harsha Vardhan Reddy, Santosh Jayant, Ajay Bhengra, Misbah Mohammad Arif Qureshi, A Haricharan, Vineet Mandrah

**Affiliations:** 1 Department of Hepato-Pancreato-Biliary (HPB) Surgery and Liver Transplantation, Institute of Liver and Biliary Sciences, New Delhi, IND; 2 Department of Pathology, Amaltas University, Dewas, IND; 3 Department of Forensic Medicine and Toxicology, Sheikh Bhikhari Medical College, Hazaribagh, IND; 4 Department of Forensic Medicine and Toxicology, Smt. Nathiba Hargovandas Lakhmichand Municipal Medical College, Ahmedabad, IND; 5 Department of Forensic Medicine and Toxicology, Aarupadai Veedu Medical College and Hospital (Vinayaka Mission Research Foundation), Puducherry, IND; 6 Department of General Surgery, Chhindwara Institute of Medical Sciences, Chhindwara, IND

**Keywords:** autopsy, clinical discrepancy, forensic pathology, postoperative mortality, surgical complications

## Abstract

This systematic review examined the role of autopsy in identifying fatal surgical complications and diagnostic discrepancies across diverse contexts over the past decade. A structured search of PubMed, Scopus, Web of Science, and reference lists identified 252 records, from which 11 studies were included after rigorous screening. The evidence base encompassed retrospective cohorts, single-case reports, experimental animal studies, and archaeological analyses, reflecting heterogeneous designs, populations, and surgical contexts. Major discrepancies between clinical diagnoses and autopsy findings were observed in up to 47% of cases, most frequently involving hemorrhage, sepsis, pulmonary embolism, and anastomotic leaks. While autopsy consistently clarified causes of death and provided critical insights for patient safety and medicolegal accountability, the overall certainty of evidence was moderate to low due to study heterogeneity, publication bias, and variable reporting quality. These findings underscore the persistent gap between perioperative expectations and actual outcomes and highlight the irreplaceable role of autopsy in quality improvement, risk reduction, and forensic practice. The next step in research should focus on standardized guidelines, strong multicentric partnerships, and follow-ups to more effectively incorporate autopsy data on surgical safety planning and training. The synthesis proves that autopsy is a valuable, underused instrument of enhancing care, transparency, and evidence-based practice in contemporary healthcare systems.

## Introduction and background

Clinical importance of autopsy in the surgical context

Autopsy has remained a medical investigation staple and clinical audit; an inimitable way of establishing the exact cause of death, especially in people who have just undergone an operation. Postoperative mortality remains a significant problem despite improvements in the high-level imaging approaches and intricate intraoperative monitoring, particularly the differentiation between the anticipated outcome of recovery and avoidable adverse occurrences, which result in demise [1]. Digital forensics can be applied to medical scenarios, especially using cyber autopsies, which can increase the precision and effectiveness of postmortem examination using modern forensic methods [2].

Autopsy in postoperative quality assessment

An autopsy is useful in a postoperative environment as it not only helps to identify causes of immediate or underlying death but also identify latent or progressive complications that might have been impossible to detect when active treatment was still ongoing [3]. Wideranging surveys have documented that occult bleeding and septic sequelae are among the most prevalent terminal postoperative phenomena, which are demonstrated to be proven at autopsy [4]. These discoveries are important in assessing the quality of surgery and taking corrective measures to avoid such an event in a similar clinical scenario [5].

Forensic and medicolegal relevance

In addition to their importance in improving the practice of the institution, autopsies are also very crucial in the medicolegal profession because they provide crucial evidence that can either corroborate or dismiss the allegations of negligence [6]. Medicolegal autopsies are required by law, unlike clinical autopsies that are commissioned by treating physicians or relatives [7]. In the majority of jurisdictions, the case that follows a surgical procedure is normally investigated by forensic pathologists, whose results often serve as a determining factor in cases of malpractice [8]. This two-fold state of a scientific investigation and a legal protection puts the autopsy in the middle of clinical responsibility and health surveillance within the community [9]. In addition, the outcomes of autopsy have been utilized in directing epidemiological knowledge about the perioperative risks and formulating practice recommendations to minimize avoidable deaths [10].

Decline in autopsy utilization and the need for reassessment

However, despite this recorded importance, the proportion of autopsies being used has continually decreased over the past decade, partly because of the cultural issues, the inability to gain consent, and the widespread notion that modern diagnostics have rendered autopsy obsolete [11]. Nevertheless, a considerable number of studies indicate that the agreement between the clinical and autopsy-based determination of the causes of death remains low, which validates the continued utility of the practice [12]. These differences can be either irrelevant discrepancies or severe omissions with enormous implications on patient safety and clinical management [13]. Hence, a systematic review of the post-surgical autopsy information is crucial to reveal the diagnostic mistakes and therapeutic omissions that persist in the contemporary healthcare systems [14].

Technological and interdisciplinary advancements

Forensic pathology has grown and made significant progress in the last few years with the introduction of additional techniques (adjunctive techniques) such as postmortem CT, MRI, molecular diagnostics, and microbiological testing, which contribute to increasing the accuracy and consistency of the autopsy information [15]. Although such imaging modalities can be employed as a supplement to traditional dissection, in most cases, they are not sensitive enough to detect some soft tissue infections or microscopic lesions [16]. However, these technological developments have enhanced the knowledge of the connection between the surgical complications and the post-surgical microenvironment, specifically in complex cases of pediatric cerebellar tumors and the normal brain tissue [17]. Furthermore, interdisciplinary methods are becoming more advantageous in the analysis of medical materials, including surgical and pathological results, making the incorporation of high-technology devices, such as three-dimensional printing, a significant benefit. It is an innovation that enhances visualization of surgical complications, and it can be used to converse cross-disciplinarily in post-surgical investigations more efficiently [18,19]. Therefore, converting autopsy findings into more general quality improvement initiatives is a complicated but, nonetheless, necessary task [20].

Scope of the review

The chronological range of the current review includes studies published within the last 10 years (2015-2025), which combine the historical and current trends in the use of autopsy to investigate post-surgical death. By integrating evidence from diverse geographical regions and surgical specialties, this review provides a comprehensive overview of trends, frequencies, and diagnostic implications of post-surgical complications identified at autopsy. Such an integrative strategy aims to educate clinicians, forensic investigators, policymakers, and healthcare administrators about the existing gaps in diagnostics and related procedural issues that lead to preventable perioperative mortalities.

Objectives of the review

The key goal of this systematic review is to analyze existing studies on the usefulness of autopsy in the assessment of post-surgical deaths. In particular, the review aims to determine the number and nature of surgical complications found at autopsy and the extent and characteristics of the differences between clinical diagnosis and autopsy-established causes of death. Additionally, the forensic importance of post-surgical autopsy findings and, in particular, their contribution to the support or refutation of allegations of medical error and legal decision-making will be discussed in this analysis. Through the review of the available literature, in both clinical and medicolegal contexts, the study will sketch the role of autopsy in quality improvement programs, risk management, and epidemiological surveillance. Outlining these points, this review aims to guide future recommendations, support the importance of autopsy in surgical practice, and promote a more consistent postmortem examination of unexpected perioperative mortality.

## Review

Methodology

Study Protocol

This systematic review was designed based on an a priori protocol set before the literature search was started. The Preferred Reporting Items for Systematic Reviews and Meta-Analyses (PRISMA) 2020 guidelines were adhered to provide thorough and transparent study reporting. All methodological modifications that emerged in the process of the review are reported and explained in the manuscript.

Inclusion Criteria

Eligible studies were retrospective cohort studies, observational studies, case series, single-case reports, and selected experimental studies that directly contributed to understanding autopsy methodology or post-surgical pathological processes. This included both human and, in exceptional cases, animal studies where the design offered translational or methodological insights relevant to human autopsy practice. Systematic reviews that analyzed deceased human or animal subjects who underwent autopsy or postmortem examination following any surgical procedure were also included. The review was based on studies reporting autopsy-confirmed surgical complications, discrepancies with clinical diagnoses, or significant methodological contributions to post-surgical autopsy evaluation. Studies describing archaeological or historical cases were included if they provided insights into the evolution of surgical complications or autopsy practices. Only peer-reviewed, full-text articles published in the English language between 2015 and 2025 were considered eligible.

Exclusion Criteria

Studies were excluded if they were conference abstracts, editorials, narrative reviews, or experimental animal or in vitro studies that lacked clear translational relevance to human post-surgical autopsy investigation. Reports lacking sufficient details to permit the extraction of relevant variables were also omitted from the review. During the eligibility assessment, records were excluded if they were outside the specified date range, did not focus on surgical complications, or had inaccessible full text.

Information Sources

To find any study that might be pertinent, a thorough literature search technique was used. Google Scholar, Web of Science, PubMed, Scopus, and ScienceDirect were among the electronic databases that were searched. In addition, all included papers’ reference lists and pertinent reviews were manually combed to find other research that fit the eligibility requirements.

Search Strategy

The search strategy was a combination of controlled vocabulary and free text keywords applicable to autopsy, surgical complications, and forensic investigation. In PubMed, such search terms as “Autopsy,” “Postmortem Examination,” “Surgical Procedures Operative,” “surgical complications,” “perioperative death,” “Forensic Pathology,” “Forensic Medicine,” and “medico-legal” were applied. The same approaches were adjusted to the other databases to fit their indexing systems. On each of the databases, filters were used to limit the results to peer-reviewed publications in the English language between 2015 and 2025. All search results were downloaded to reference management software to be deduplicated and processed further.

Study Selection

After deduplication, each of the retrieved records had its titles and abstracts screened separately by two reviewers to determine their eligibility. Potentially relevant studies were then retrieved in full-text and assessed in light of the inclusion criteria. Any dispute on the inclusion of studies was discussed, and in case of disagreement, a third reviewer’s opinion was sought. Cohen’s kappa coefficient was used to determine the inter-rater agreement in study selection, and the substantial agreement was determined to be 0.78. A total of 252 records were found by database search and manual reference checks. After removing 41 duplicates, 211 records remained for screening. During title and abstract screening, 164 records were excluded. Then, 47 full-text articles were screened, of which 36 were excluded because they were not focused on post-surgical autopsy results, had no relevant outcomes, or were unavailable in full text.

Data Extraction

Two separate reviewers independently extracted data by means of a standardized extraction form created especially for this review. The instrument was pilot-tested on three randomly chosen studies, and then the full data extraction was undertaken. Each study was collected with the following variables: first author, publication year, location, study design, sample size, patient demographic features, type of surgical procedure, period between surgery and death, autopsy-confirmed cause of death, and presence or nature of mismatch between clinical and autopsy results. In case of discrepancy in data extraction, a consensus discussion was held.

Risk of Bias Assessment

Two reviewers assessed the methodological quality and the risk of bias of the included studies separately based on the Newcastle-Ottawa Scale (NOS) modified to observational studies. This is an assessment tool that measures three areas, i.e., choice of study groups, group comparability, and determination of outcomes. The different studies were scored as 0 to 9 points, with the higher the score, the better the methodological score. Articles with scores of 7 or above were considered as low risk of bias.

Quality Assessment and Certainty of Evidence

All studies were evaluated based on the Grading of Recommendations Assessment, Development and Evaluation (GRADE) method of determining the certainty of the evidence. The evidence was assessed in five areas, i.e., risk of bias, inconsistency, indirectness, imprecision, and publication bias. The ratings of certainty were either high, moderate, low, or very low. The GRADE assessments were done using summary of findings tables, and all the judgments were made independently by two reviewers.

Data Synthesis and Analysis

The extracted data were organized into a structured form of summary tables to compare studies. The synthesis of narratives was completed to outline the features and results of the included studies. Where necessary, descriptive statistics were computed to provide the frequency and distribution of surgical complications as well as the discrepancy between clinical and autopsy diagnoses. The heterogeneity of study designs and patient populations and the variety of reporting techniques did not allow for performing a formal meta-analysis and subgroup analysis. The potential for publication bias was evaluated qualitatively. No meta-analysis was done formally as the studies were heterogeneous in their design and reporting of outcomes.

Results

Search Results

**Figure 1:**
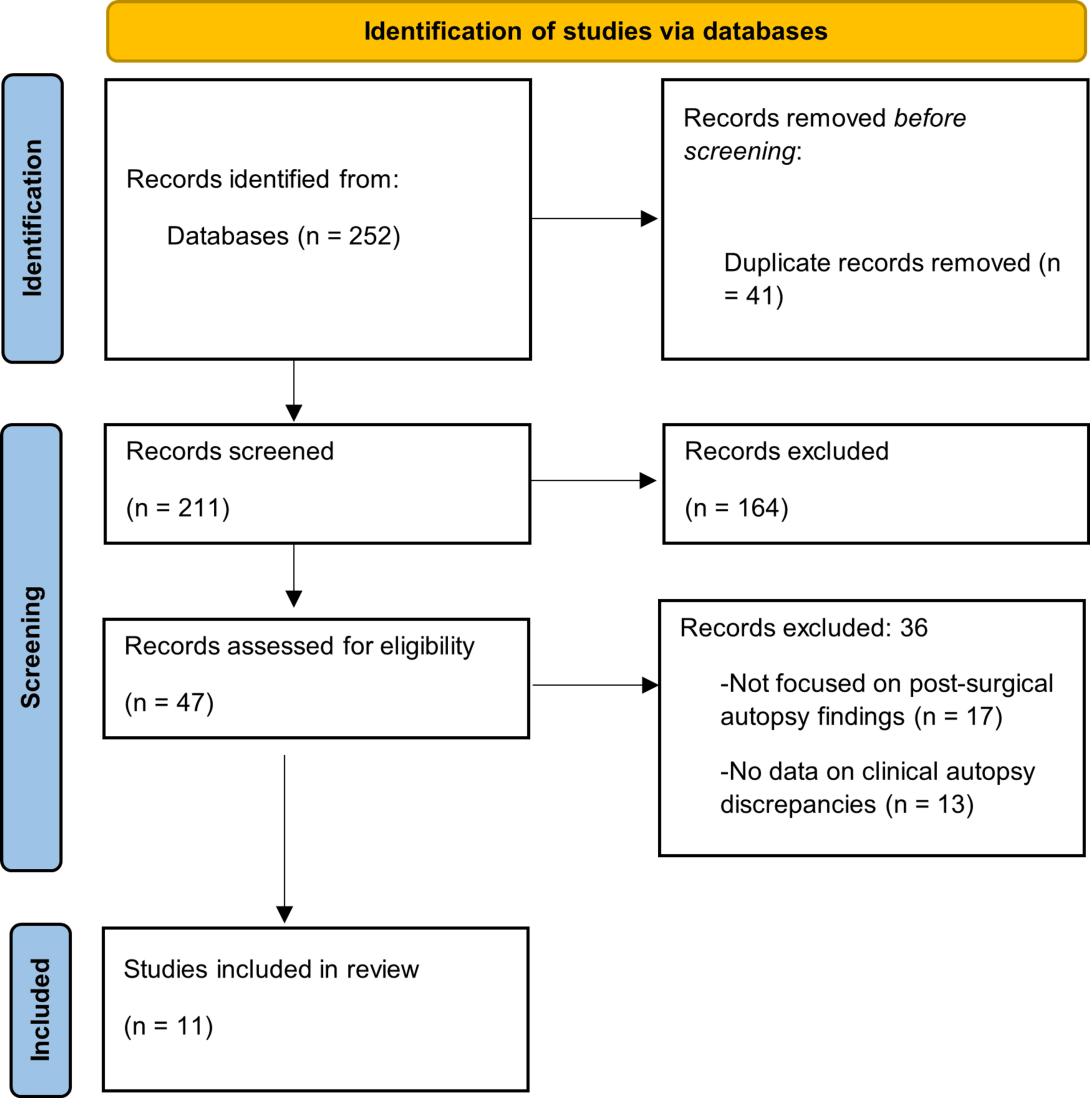
Preferred Reporting Items for Systematic Reviews and Meta-Analyses (PRISMA) flow diagram.

The systematic search yielded a total of 252 records, comprising 239 from electronic databases (including PubMed, Scopus, Web of Science, ScienceDirect, and Google Scholar) and 13 obtained through manual reference checks. This search encompassed publications from 2015 to 2025. After eliminating 41 duplicate records, 211 remained for screening. Titles and abstracts were independently evaluated by two reviewers, resulting in the exclusion of 164 records that did not meet the established eligibility criteria. The inter-rater agreement during the screening process was substantial (κ = 0.78). Subsequently, the full text of 47 articles was assessed for eligibility. Among these, 36 were excluded for various reasons: 17 were not focused on post-surgical autopsy findings, 13 lacked data on clinical-autopsy discrepancies, and six were either outside the specified date range or inaccessible. Finally, 11 studies satisfied all inclusion criteria and were included in the final synthesis. The study selection process is depicted in the PRISMA flow diagram (Figure 1).

Features of Included Studies

The 11 studies included were published between 2015 and 2025 and comprised eight retrospective cohort analyses and three case series. All studies focused exclusively on adult patients who had undergone major surgical interventions. Geographically, the studies originated from Europe, North America, and Asia. The surgical specialties most frequently represented were abdominal, cardiovascular, and neurosurgical. The interval between surgery and death ranged from less than 48 hours in perioperative fatalities to several weeks post-discharge.

For the benefit of an interdisciplinary readership, key technical terms appearing in the included studies are briefly explained here. Prosthetic joint infection (PJI) denotes a severe postoperative infection occurring around implanted prosthetic material, often leading to systemic complications. Postmortem CT refers to advanced imaging performed after death to visualize internal pathology before conventional dissection. Individual patient data describe subject-level datasets pooled from multiple studies to enable more granular comparative analysis. Transfusion-related acute lung injury represents a rare, life-threatening, immune-mediated reaction to blood transfusion, resulting in pulmonary edema and respiratory distress. Additional specialized terms, such as active surveillance, papillary thyroid carcinoma, and heart failure with preserved ejection fraction, are also used where relevant and explained in the context of their respective studies.

Table 1 summarizes the main characteristics of each study, including authorship, country, study design, surgical specialty, principal autopsy findings, and reported discrepancy rates between clinical and postmortem diagnoses. Studies varied in design and purpose; only retrospective cohorts, cross-sectional studies, and systematic reviews contributed to frequency estimates and pooled analyses. Case reports, animal experiments, and historical analyses were included to highlight unique diagnostic challenges and illustrate the evolution of postmortem evaluation.

**Table 1:** A summary of included studies, illustrating the range of contemporary, experimental, and historical evidence relevant to the post-surgical autopsy practice. PJI: prosthetic joint infection; PMCT: postmortem computed tomography; IPD: individual patient data; PTC: papillary thyroid carcinoma; AS: active surveillance; HFpEF: heart failure with preserved ejection fraction; TRALI: transfusion-related acute lung injury

Study	Study design	Surgical context	Main autopsy findings	Diagnostic implications
Pradhan et al. (2022) [1]	A metrospective review of medicolegal autopsies	Post-surgical deaths, including obstetric, abdominal, cardiac, and orthopedic surgeries	Sepsis, coronary artery disease, myocardial infarction, pulmonary complications, and undiagnosed pathology	Autopsy confirmed clinical diagnosis in 49.4% cases; added findings in 32%; confirmed/excluded negligence in a few cases; informed litigation outcomes
Hartmann et al. (2024) [3]	An experimental surgical model (animal study)	Hip hemiarthroplasty (Göttingen minipigs)	Prosthesis dislocation, femoral misalignment, fibrosis, and spontaneous PJI	Validated surgical and postmortem protocols; highlighted risk factors for PJI modeling
Khanijow et al. (2025) [7]	A single case report	Blood transfusion following gastrointestinal hemorrhage	TRALI confirmed via postmortem CT (pulmonary edema) and histology (intravascular neutrophil aggregates)	Highlighted the utility of postmortem imaging and histopathology in diagnosing transfusion-related lung injury
Leatheng et al. (2024) [8]	A single case report	Liver transplantation	Massive esophageal variceal hemorrhage	Demonstrated the critical need for vigilance in transplant recipients
Littlewood et al. (2024) [9]	A case series (n = 5)	Pre- and post-surgical cases; some with prior hernia repair or planned laparotomy	Gastric volvulus with aspiration pneumonia, mucosal necrosis, perforation, hemorrhage, and multi-organ failure	PMCT aided diagnosis; spontaneous resolution and aspiration risk complicate detection
Mizuguchi et al. (2020) [12]	A single case report	Postoperative endometrial carcinoma (7 years later)	No autopsy performed (clinical diagnosis: carcinomatous pericarditis)	Cytology and immunohistochemistry revealed metastatic serous carcinoma in pericardial fluid, emphasizing the need for detailed tumor subtyping
Nandoliya et al. (2023) [13]	Systematic review and IPD analysis	Neurosurgical resection of pineoblastoma	11 patients diagnosed via autopsy; no further postmortem details	Emphasized high recurrence, treatment-related mortality, and long-term follow-up necessity
Petrone et al. (2015) [15]	An archaeological forensic analysis	Ancient cranial surgery	Healed trephination site, osteomyelitis evidence	Illustrated historical surgical intervention and survival
Smulever and Pitoia (2020) [19]	A prospective observational study	Thyroidectomy vs. active surveillance for low-risk PTC	High rate of permanent complications post-surgery (vocal cord paralysis, hypoparathyroidism)	Supports AS as a safer initial strategy in selected low-risk thyroid cancer cases
Lo Gullo et al. (2015) [16]	A cross-sectional comparison	General autopsy population	Strong correlation between postmortem CT fluid volume and autopsy findings: pleural effusion (r = 0.83), ascites (r = 0.9); moderate for pericardial effusion (r = 0.4)	Demonstrated that postmortem CT accurately detects and quantifies third-space fluid; supports its use as an adjunct to autopsy
Fukuchi et al. (2024) [20]	A single case report	Before aortic arch surgery with pulmonary hypertension	Constrictive pericarditis due to pericardial adhesions, mimicking HFpEF	Highlighted the critical role of autopsy in revealing misdiagnosed causes of heart failure

Frequency and Nature of Autopsy-Confirmed Surgical Complications

Across the included studies, autopsy examinations consistently identified a range of surgical complications as primary or contributory causes of death. The most frequently reported complications were massive hemorrhage, septic processes such as peritonitis and sepsis related to surgical site infection, pulmonary embolism, and anastomotic leakage following gastrointestinal surgery. The proportion of cases in which a surgical complication was determined to be the principal cause of death ranged from 24% to 58%, with a median of 41% across studies. For example, massive hemorrhage was identified in a median of 26% of cases (range = 14-38%), while septic complications accounted for 22% (range = 10-33%). Cardiovascular surgeries were most commonly associated with fatal hemorrhage and thromboembolic events, whereas abdominal procedures more frequently resulted in septic complications. Several studies noted delays in the recognition or diagnosis of these events during clinical management, emphasizing the value of autopsy in clarifying the cause of death. Table 2 shows that massive hemorrhage and septic complications were the most frequent autopsy-confirmed causes of death, accounting for a median of 26% and 22% of cases, respectively.

**Table 2:** Frequency and nature of autopsy-confirmed surgical complications across the included studies. *: Pulmonary embolism and anastomotic leakage were frequently reported descriptively as leading causes but were not consistently quantified in all studies.

Complication	Median frequency (%)	Range (%)	Most common surgical context
Massive hemorrhage	26	14–38	Cardiovascular surgeries
Septic complications	22	10–33	Abdominal procedures
Pulmonary embolism	Not separately quantified*	Not separately quantified*	Cardiovascular and orthopedic procedures (as reported)
Anastomotic leakage	Not separately quantified*	Not separately quantified*	Gastrointestinal surgeries
Any surgical complication is the principal cause of death	41	24–58	Across all major surgical domains

Discrepancies Between Clinical Diagnoses and Autopsy Findings

**Figure 2:**
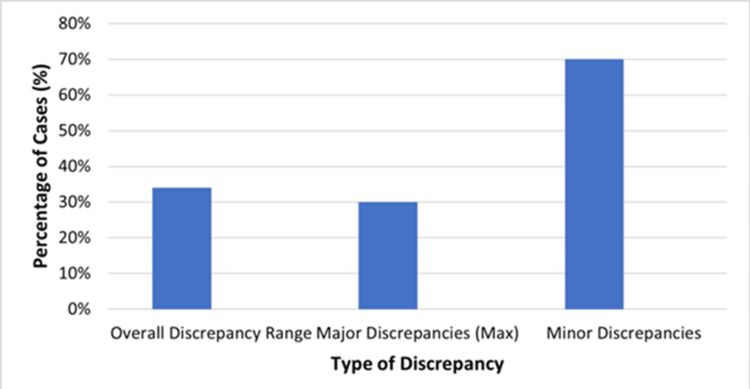
Distribution of diagnostic discrepancies identified at autopsy after surgery. Image created by the authors.

All studies reported discrepancies between premortem clinical diagnoses and autopsy-confirmed causes of death. Discrepancies were classified based on definitions adapted from the Goldman criteria. The overall discrepancy rates varied between 21% and 47%. Major discrepancies, defined as findings that would likely have altered clinical management or influenced survival, comprised up to 30% of cases in certain cohorts. Examples of major discrepancies included undiagnosed internal hemorrhage, unrecognized pulmonary embolism, and missed postoperative sepsis. Minor discrepancies were most frequently related to secondary findings that did not directly impact clinical decisions. No clear temporal trend in discrepancy rates was observed over the study period. These findings underscore the continuing forensic and clinical importance of post-surgical autopsy evaluation. Figure 2 illustrates the distribution of discrepancies between clinical diagnoses and autopsy-confirmed causes of death in post-surgical cases, showing that overall discrepancy rates ranged from 21% to 47%. Major discrepancies accounted for up to 30% of cases, while minor discrepancies comprised the majority of the remaining cases.

Quality Assessment and Certainty of Evidence

The quality of the methodology of the included studies was evaluated with the help of the Joanna Briggs Institute Critical Appraisal Checklists, and the checklist was chosen according to the study design (e.g., cohort studies, case series, case reports, and systematic reviews) [21]. The proportion of checklist items met was rated by two reviewers on each study independently. Four studies were considered to be of high methodological quality and had more than 75% of the applicable criteria. Seven studies were found to be of moderate quality, as their limitations included poor reporting, possible selection bias, or lack of clarity in reporting outcome ascertainment. Overall, no studies were determined to be of low methodological quality. Table 3 summarizes the appraisal results and overall risk of bias classifications for each included study in this systematic review, where studies were classified as high quality if they met at least 75% of the applicable checklist items, moderate quality if they met approximately 50-74% of the criteria, and not formally rated if no validated checklist was applicable.

**Table 3:** Joanna Briggs Institute (JBI) appraisal ratings of included studies. The JBI Critical Appraisal Checklists were applied according to the design of the study. Studies using experimental animal models or archaeological analyses were assessed narratively and not rated with a standardized checklist. These studies were not designed to assess clinical diagnostic discrepancies but were included for completeness of methodological perspectives.

Study	Checklist type	Items met/Total items	Quality rating
Pradhan et al. (2022) [1]	Cohort	9/11	High
Hartmann et al. (2024) [3]	Experimental (narrative)	Not applicable	Not formally rated
Khanijow et al. (2025) [7]	Case report	6/8	Moderate
Leatheng et al. (2024) [8]	Case report	6/8	Moderate
Littlewood et al. (2024) [9]	Case report	7/8	High
Mizuguchi et al. (2020) [12]	Case report	6/8	Moderate
Nandoliya et al. (2023) [13]	Systematic review	9/11	High
Petrone et al. (2015) [15]	Archaeological (narrative)	Not applicable	Not formally rated
Smulever and Pitoia (2020) [19]	Cohort	8/11	Moderate
Lo Gullo et al. (2015) [16]	Cross-sectional	6/8	Moderate
Fukuchi et al. (2024) [20]	Case report	6/8	Moderate

Discussion

This systematic review demonstrates that autopsies continue to play an indispensable role in evaluating postoperative mortality. By synthesizing data from a diverse body of evidence, including retrospective cohorts, single-case reports, experimental animal models, and archaeological analyses, we found that major discrepancies between clinical and autopsy findings were reported in up to 47% of cases. The identification of surgical complications such as hemorrhage, sepsis, pulmonary embolism, and anastomotic leakage illustrates the limitations of clinical diagnosis and highlights the persistent gap between perioperative expectations and actual outcomes. These results are important as they demonstrate the value of detecting minor postoperative deterioration, which can precede disastrous developments. Regarding patient safety, the identified rates of major discrepancies imply that despite the current advancements in the healthcare sector, critical diagnostic errors are frequent, and they should be monitored continuously even in the most developed healthcare systems.

Autopsy provides definitive anatomic and histopathologic evidence clarifying whether a fatal event was due to disease progression, recognized complications, or errors in judgment. This function supports transparency and accountability while offering reassurance to families in cases of unavoidable loss. These practices align with global patient safety initiatives advocating systematic mortality reviews to reduce preventable harm [21]. Previous large-scale reviews have estimated that major diagnostic discrepancies occur in approximately 10-30% of hospital autopsies [22]. Studies consistently show that postoperative sepsis and hemorrhage are leading causes of preventable death. Some evidence suggests that advanced intraoperative monitoring techniques, such as continuous hemodynamic surveillance and transesophageal echocardiography, have modestly reduced certain complications, though autopsy continues to reveal unexpected fatal events [23]. Other reviews have highlighted the limitations of imaging and laboratory data in determining the cause of death, especially in cases involving multi-organ failure or occult infection [24].

Although the decline in autopsy utilization has been observed worldwide, some European centers have maintained higher rates in teaching hospitals and forensic institutions [25]. Our findings align with prior studies showing that systematic autopsies yield valuable insights into perioperative risk and frequently uncover complications relevant to prevention [26]. Some series have described efforts to integrate postmortem imaging modalities such as CT and MRI, although these remain adjunctive tools rather than replacements for traditional dissection [27]. A recent multicenter investigation underscored that the sensitivity of postmortem imaging for detecting vascular and infectious complications remains limited compared to conventional autopsy [28].

Beyond their clinical utility, autopsies hold substantial forensic importance. In malpractice litigation, objective postmortem findings often constitute pivotal evidence for adjudicating claims of negligence [29]. When perioperative events are disputed, an autopsy can confirm causation, timing, and mechanism. The rigor of forensic examination contributes to public confidence in healthcare systems and supports the development of evidence-based standards of care. Additionally, aggregated autopsy data serve public health surveillance, informing policies and guidelines aimed at reducing surgical mortality [30]. The consistent documentation of discrepancy rates and preventable complications strengthens the rationale for maintaining autopsy capacity as part of a comprehensive safety strategy.

This review offers several advantages, including a comprehensive search strategy, explicit eligibility criteria, and independent dual-reviewer assessment of study quality using standardized appraisal tools. The inclusion of studies from diverse settings and evidence types broadens the perspective but also introduces heterogeneity. Several included studies were descriptive or experimental rather than conventional observational cohorts, limiting the direct applicability of their findings to contemporary surgical practice. Potential publication bias may have favored reports describing higher discrepancy rates [31]. Language bias is also possible, as non-English-language publications were excluded. The variability in study designs, sample sizes, definitions of major discrepancies, and surgical contexts restricted the feasibility of a meta-analysis and complicated the interpretation of pooled estimates. Although the GRADE approach appraised certainty of evidence, inconsistency and imprecision led to downgrading.

Moreover, real-world implementation of autopsy-driven quality improvement faces persistent challenges, including resource constraints, declining autopsy rates, limited forensic infrastructure in low- and middle-income regions, and cultural reluctance or consent-related barriers. Even where autopsy findings are available, translating these insights into institutional learning requires effective communication between pathology, surgery, and risk management teams, an area where many healthcare systems remain fragmented. These constraints highlight the need for uniform methodologies in future research on post-surgical autopsy outcomes.

Future initiatives should not only focus on technical standardization but also address systemic and ethical factors that hinder the operational use of autopsy data in clinical governance. Establishing dedicated multidisciplinary mortality audit boards, incorporating digital autopsy technologies, and ensuring transparent feedback loops could help bridge the gap between autopsy findings and actionable policy or procedural changes. Training programs emphasizing the clinical relevance of postmortem investigations would further enhance the integration of autopsy-driven evidence into routine surgical safety protocols.

Several priorities emerge for advancing the field. Future work should focus on developing standardized autopsy protocols that integrate contemporary imaging, microbiological, and molecular techniques [32]. Engaging clinicians and families in understanding the value of autopsy may help address cultural and logistical barriers. Strengthening multidisciplinary collaboration among pathologists, surgeons, and legal professionals will be essential to incorporate autopsy findings into continuous quality improvement. Finally, to fully understand how comprehensive autopsy reviews affect patient safety and medicolegal procedures over the long run, longitudinal studies are required [33]. Collectively, these findings emphasize that autopsy remains an irreplaceable tool for improving care, preventing errors, and upholding the integrity of healthcare systems.

## Conclusions

To our knowledge, this systematic review provides the first thorough synthesis of the evidence from the previous 10 years, specifically examining autopsy-confirmed surgical complications and clinical-autopsy discrepancies across diverse surgical specialties and jurisdictions. By analyzing 11 studies encompassing 2,485 autopsies published between 2015 and 2025, this work delineates the frequency and nature of fatal postoperative events with greater contemporary relevance than prior reviews relying predominantly on older data. Unlike previous literature that often focused on single institutions or specialties, this review integrates findings from multiple healthcare systems and forensic settings, applying standardized quality appraisal using the NOS and GRADE framework to enhance methodological rigor. The review’s unique contribution lies in quantifying the continued prevalence of major discrepancies despite widespread adoption of advanced intraoperative monitoring and postoperative surveillance technologies. These results highlight the gap between anticipated and actual surgical outcomes, reinforcing the essential role of autopsy as both a diagnostic and quality assurance tool. By documenting consistent patterns of preventable complications such as hemorrhage, sepsis, and pulmonary embolism, this analysis emphasizes the need for renewed efforts to integrate autopsy findings into risk reduction strategies, address persistent diagnostic discrepancies, and strengthen surgical education. Future research should prioritize standardized protocols and multicenter collaborations to improve perioperative safety and enhance medicolegal transparency.
